# One-step generation of triple gene-targeted pigs using CRISPR/Cas9 system

**DOI:** 10.1038/srep20620

**Published:** 2016-02-09

**Authors:** Xianlong Wang, Chunwei Cao, Jiaojiao Huang, Jing Yao, Tang Hai, Qiantao Zheng, Xiao Wang, Hongyong Zhang, Guosong Qin, Jinbo Cheng, Yanfang Wang, Zengqiang Yuan, Qi Zhou, Hongmei Wang, Jianguo Zhao

**Affiliations:** 1 State Key Laboratory of Stem Cell and Reproductive Biology, Institute of Zoology, Chinese Academy of Sciences, Beijing 100101, China; 2 University of Chinese Academy of Sciences, Beijing 100049, China; 3 State Key Laboratory of Brain and Cognitive Sciences, Institute of Biophysics, Chinese Academy of Sciences, Beijing 100101, China; 4 Institute of Animal Sciences, Chinese Academy of Agricultural Sciences, Beijing 100193, China; 5 State Key Laboratory of Brain and Cognitive Sciences, Institute of Biophysics, Chinese Academy of Sciences, Beijing 100101, China

## Abstract

Pig shows multiple superior characteristics in anatomy, physiology, and genome that have made this species to be more suitable models for human diseases, especially for neurodegenerative diseases, because they have similar cerebral convolutions compared with human neocortex. Recently, CRISPR/Cas9 system shows enormous potential for engineering the pig genome. In this study, we expect to generate human Parkinson’s disease pig model using CRISPR/Cas9 system by simultaneously targeting three distinct genomic loci, parkin/DJ-1/*PINK1*, in Bama miniature pigs. By co-injection of Cas9 mRNA and multiplexing single guide RNAs (sgRNAs) targeting *parkin, DJ-1*, and *PINK1* genes, respectively, into *in vivo* derived pronuclear embryos, we simultaneously targeted three distinct genomic loci. The gene modified piglets remain healthy and display normal behavior at the age of 10 months. In addition, despite the high number of sgRNAs were employed in the present study, our trio-based whole-genome sequencing analysis suggested that the incidence of off-target events is low. Our results demonstrate that the simplicity, efficiency, and power of the CRISPR/Cas9 system to allow for the modification of multiple genes in pigs and yield results of high medical value.

Parkinson’s disease (PD) is the most common neurodegenerative movement disorder in the elderly. While most cases are sporadic, about 5–10% of people are now known to have forms of the disease that occur because of a mutation of one of several specific genes^1^. Recessively inherited loss-of-function mutations in the *DJ-1, parkin*, and *PINK1* genes are linked to familial cases of early-onset PD^2^,^3^,^4^. However, neither single nor triple knockout mice lacking *DJ-1, parkin*, and *PINK1* genes can recapitulate the clinical symptoms of idiopathic or inherited PD^5^,^6^. The lack of a proper model has hindered our ability to develop therapies against human PD. Recently, numerous pig models of neurodegenerative disorders, such as amyotrophic lateral sclerosis (ALS)^7^ and Huntington’s disease (HD)^8^, have been developed using gene engineering approaches because pigs share many physiological similarities with humans. To understand the pathophysiology of PD, and to develop novel therapies for improved symptomatic management, we were therefore prompted to generate *DJ-1, parkin*, and *PINK1* genes modified pigs to attempt to obtain the relevant disease models of PD.

Recently, through the use of the CRISPR/Cas9 system, biallelic gene knockout pigs were efficiently generated in a single step through a direct cytoplasmic injection of Cas9 mRNA and sgRNA into pig zygotes^9^,^10^. These findings indicated that the CRISPR/Cas9 system shows enormous potential in establishing large animal models of neurodegenerative diseases by engineering genome in spite of the lack of embryonic stem cell lines for genomic manipulation^11^. Nevertheless, the versatile functionalities of the CRISPR/Cas9 system, such as multiplexed genome editing, remain to be developed and improved in pigs. This requirement is particularly important when simultaneous modification of multiple genes functioning in concert is needed to obtain a desired phenotype. For example, inactivation of all three recessive PD genes, *DJ-1, parkin*, and *PINK1*, may have synergistic effects and cause more severe PD pathology than single gene inactivation, which may explain why patients with digenic *parkin* and *PINK1* mutations had a lower age of onset than those with single mutation^12^. Thus, it drives us to recapitulate some key features of PD disease by generating *DJ-1/parkin/PINK1* triple-gene modified pigs using CRISPR/Cas9 system.

## Results

**Figure 1:**
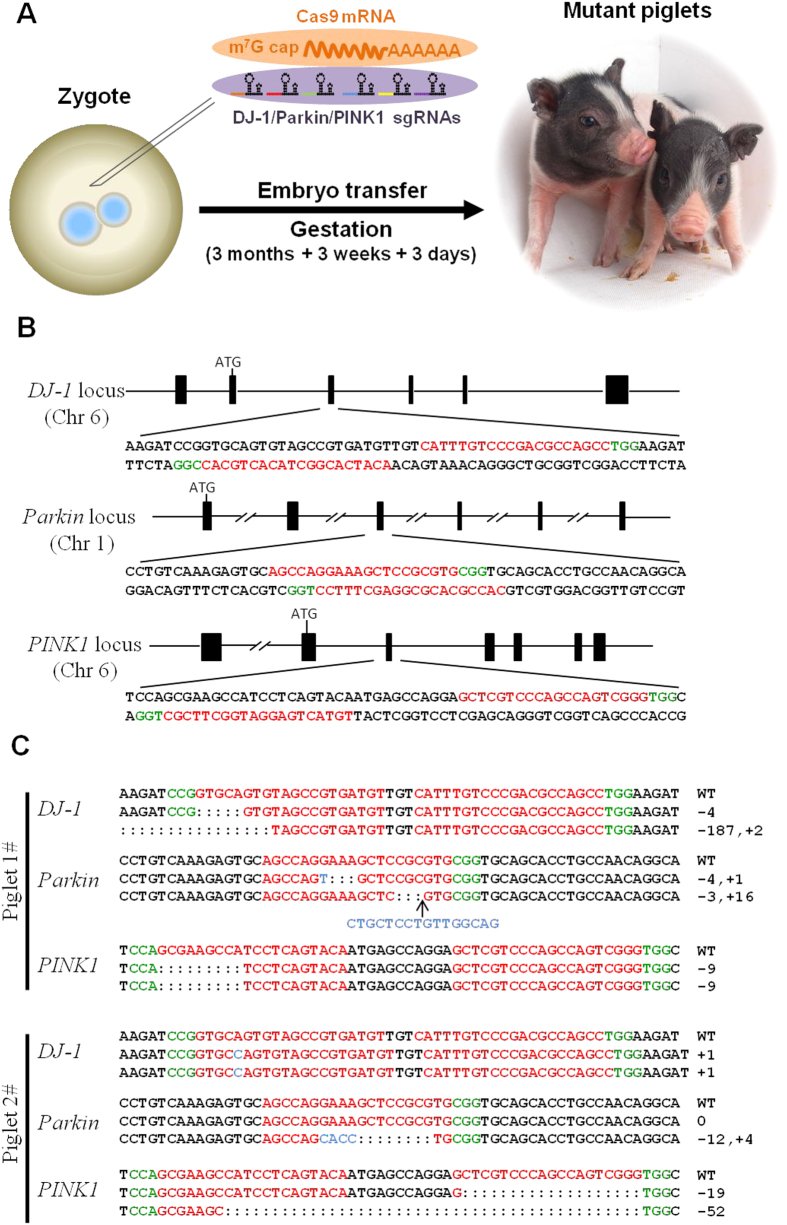
Generation of *DJ-1*/*parkin*/*PINK1* triple gene-modified pigs using the CRISPR/Cas system. (**A**) Schematic diagram of generation of triple gene targeted pigs by zygote injection of Cas9 mRNA/sgRNAs. *In vitro* transcribed Cas9 mRNA and multiplexing sgRNA were co-injected into the cytoplasm of one-cell stage pig embryos. Then, the injected embryos were transferred into recipient gilts to produce the genetically modified offspring. (**B**) Schematic diagram of sgRNAs targeting at *DJ-1, parkin* and *PINK1* locus. The PAM sequences are highlighted in green. The sgRNA targeting sites are highlighted in red. (**C**) Sanger sequencing of the targeting site in mutant pigs. For each gene, the wild-type sequence is shown at the top with the target sites highlighted in red. At least 15 TA clones of the PCR products were analyzed by DNA sequencing. The change in length caused by each mutation is to the right of each sequence. The PAM sequences are highlighted in green; the mutations in blue; deletions (−), insertions (+).

In the present study, we produced pigs with mutations in multiple targeted genes. By co-injection of Cas9 mRNA and multiplexing sgRNAs into *in vivo* derived pronuclear embryos, we simultaneously targeted three distinct genomic loci in Bama miniature pigs (Fig. 1A).

The simultaneous use of two adjacent sgRNAs targeting one locus significantly increased the targeting efficiency and improved Cas9-mediated genome targeting^13^,^14^. Hence, we designed six sgRNAs targeting six different genomic sites encoding pig DJ-1 (sgDJ1-1 and sgDJ1-2), parkin (sgparkin-1 and sgparkin-2) and *PINK1* (sgPINK1-1 and sgPINK1-2) (Fig. 1B). The sgRNAs were designed to contain 20-nucleotide customized spacer sequences together with tracrRNA-derived sequences as previously described^15^. A mixture of sgDJ1-1, sgDJ1-2, sgparkin-1, sgparkin-2, sgPINK1-1 and sgPINK1-2 (six sgRNAs) with Cas9 mRNA was injected into one-cell-stage pig embryos. Next, we transferred the injected embryos into surrogate pigs to produce piglets. A total of 34 embryos were delivered to 3 surrogates, and one of surrogates was successfully impregnated and delivered two live-born piglets (Supplementary information, Table S1). We amplified target sites from the genomic DNA of each individual piglet and analyzed the genotype by Sanger sequencing. Surprisingly, both of the founders harbored site-specific indels in the targeting sites. One piglet showed biallelic modification of all three genes, and another showed biallelic modification of the *DJ-1* and *PINK1* genes and monoallelic mutations of *parkin* gene (Fig. 1C). These results demonstrated that piglets carrying biallelic mutations in three different genes could be generated in one step through direct zygote injection with high efficiency.

**Figure 2:**
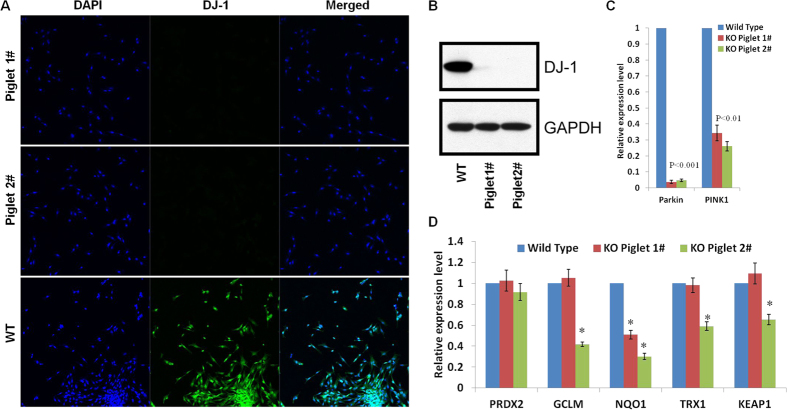
Brife phenotypic identification of triple gene-modified piglets. (**A**) DJ-1 immunofluorescence analysis of the fibroblasts of WT and two gene modified piglets. The fibroblasts of gene-modified piglets are negative for anti-DJ-1 staining; in contrast, the WT controls show positive staining in the fibroblasts. (**B**) Western blot analysis showed the absence of DJ-1 protein expression in the fibroblasts of these two gene modified piglets. (**C**) Quantitation of *parkin* and *PINK1* gene expression in the fibroblasts of WT and two gene modified piglets. *parkin* and *PINK1* gene expression was dramatically decreased in the gene modified-derived pig fibroblast cells compared with wild-type cells based on real-time RT-PCR. The graph demonstrates the results of the gene expression levels normalized against loading controls (arbitrary units, wild type = 1). (**D**) Comparison of oxidative stress defense gene expression in wild type and gene modified-derived pig fibroblast cells by real-time RT-PCR. The graph demonstrates the results of the gene expression levels normalized against loading controls (arbitrary units, wild type = 1). *p < 0.05.

We further examined whether gene modifications caused a depletion of gene expression or related phenotypes. Immunofluorescence (Fig. 2A) and western blot analysis (Fig. 2B) showed the absence of DJ-1 protein expression in the fibroblasts of these two gene modified piglets, which confirmed that both of the *DJ-1* gene alleles were inactivated. Due to the difficulty of obtaining an effective antibody to determine the expression of *parkin* and *PINK1* in pigs, real-time RT-PCR was employed. The relative *parkin* and *PINK1* gene mRNA expression was also dramatically decreased in the fibroblast cells from the two gene modified pig compared with wild-type cells based on real-time RT-PCR (Fig. 2C), most likely due to nonsense-mediated mRNA decay^16^. Furthermore, because oxidative stress and mitochondrial dysfunction have been linked to the pathogenesis of PD^17^, we also showed that the transcription of some stress defense genes was disrupted in the gene modified pig fibroblast cells (Fig. 2D). However, the two piglets remain healthy with a normal growth rate for Bama miniature pigs and typical symptoms of Parkinson’s disease have not been observed in 10-month-old live mutant pigs in this study.

**Figure 3:**
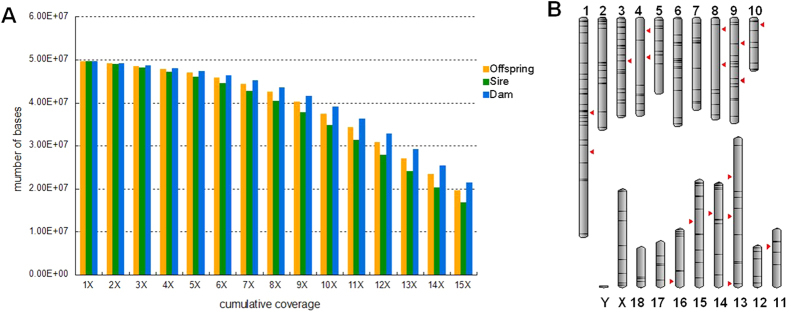
(**A**) Cumulative distribution of coverage of whole genome sequencing for three individuals. Shown is the absolute number of genomic sites in the coding sequences (CDS) achieving coverage equal or higher than the coverage indicated on the x axis. (**B**) None of indel mutations located around potential off-target regions as predicted by the CasOT program. Red triangles indicate unique de novo indels detected by whole-genome sequencing. Black bars indicate the putative off-target sites predicted by the CasOT program.

Previous studies have suggested that the CRISPR/Cas9 systems might tolerate sequence mismatches distal from the protospacer adjacent motif (PAM) at the 5′ end of sgRNAs, which would likely induce off-target mutations^18^. Specifically, a high number of sgRNAs were employed in the present study. We thus sought to test the possible off-target effects in genome-modified pigs derived from multiplexing sgRNAs-injected zygote. We performed whole-genome sequencing in the two parents and one of the piglets (Fig. 3A, Supplementary information, Figure S1) to assess the degree of mutagenesis across the entire genome. We largely focused on the identification of small indels introduced by non-homologous end joining (NHEJ)-mediated repair. After the filtering process (methods section), a set of small indels that were most likely to be true positives and potential off-target mutations (rather than the mutations that arose in the parental genomes) were detected by whole-genome sequencing. Consequently, we identified a total of 19 de novo indels in the offspring throughout the genome (Table 1). The de novo mutation frequency (6.77 × 10^−9^) is closed to that previously estimated in human (1.5 × 10^−9^)^19^, which indicates that our CRISPR/Cas9 system does not significantly increase the rate of de novo indel mutations. Further, the low de novo mutation frequency may indirectly reflect the low incidence of off-target events. Additionally, these mutations were all located in the non-coding genomic regions (Table 1), which suggests no obvious effect on the function of the protein. We further asked whether these *de novo* indels were generated spontaneously or induced by the CRISPR/Cas9 system. To this end, we performed a comprehensive prediction for potential off-target sites by a CasOT program^20^, and we found that no indel mutations located around the predicted/potential off-target regions (Fig. 3B). These results suggested that these mutations were most likely to occur spontaneously. Moreover, the potential off-target sites, which were located within the coding regions, were well covered by sequencing reads (also confirmed by Sanger sequencing, Supplementary information, Figure S2), and no *de novo* mutations were identified in these off-target sites and flanking regions (Supplementary information, Table S2). In addition, the whole-genome sequencing analysis demonstrated that the present CRISPR/Cas9 targeting did not lead to mosaic mutations (data not shown). We concluded that the CRISPR/Cas9 system may not significantly increase the incidence of off-target cleavages in the pig genome, though a high number of sgRNAs was employed in the present study. Our study is consistent with the recent reports showing off-target mutations are rare in Cas9-modified mice^21^.

## Discussion

The genetic manipulation of pigs is a crucial approach for improving livestock production and the study of human disease. However, the generation of gene modified pigs has been impeded by the slow, tedious, and expensive procedure of genetic modification due to the lack of embryonic stem cell lines. The most exciting finding in this study is the demonstration of robust multiplex, biallelic gene targeting in the pig without induction of off-target mutattions. A unique capability of the CRISPR/Cas9 system is multiplex genome engineering by delivering a single Cas9 enzyme and two or more single guide RNAs (sgRNAs) targeted to distinct genomic sites. Through the co-injection of Cas9 mRNA and the multiplexing of sgRNAs into *in vivo* derived pronuclear embryos, we simultaneously targeted three distinct genomic loci in Bama miniature pigs in a highly efficient manner.

Several previous studies have reported that simultaneous use of dual sgRNAs to target an individual gene significantly improved the CRISPR/Cas9-mediated genome targeting efficiency^13^,^14^,^22^. In line with this, we found that dual gRNAs could indeed simultaneously target three distinct genomic loci in Bama miniature pigs in a highly efficient manner. This could be because different sgRNAs have substantially variable DNA cleavage efficiency, and dual gRNAs could guarantee the CRISPR system has maximize occurrence of genetic modification at the target site. Actually, in our present study, the allelic mutations at the *parkin* target site from piglet 1# were induced by different sgRNAs. Recently, dual sgRNAs were used to induce a deletion of the chromosome sequence between the two sgRNA-targeted loci, which is a very useful technique for creating null alleles and targeting noncoding regions in the genome such as enhancers and silencers that control expression of disease-relevant genes^22^. However, Wang *et al*., reported that combination of some two sgRNAs may exhibit an antagonistic effect in targeting. Such antagonistic effect reminded us that when multiple gRNAs were used in combination, the side effect should be taken into consideration^23^.

Although CRISPR/Cas9 systems can serve as the basis of a simple and highly efficient method for performing genome editing in various organisms, they do not have perfect specificity and can induce high-frequency off-target mutagenesis at sites in the genome other than the desired on-target site^18^. The major concern about the present study is the potential off-target effect caused by high number of sgRNAs. Unlike previously reports, in which limited numbers of potential target DNA sequences with point or combined mismatches *in silico* predicted by comparison with the authentic targeting sites were tested, we performed whole-genome sequencing in the parents and one of the piglets (1#) to assess the degree of mutagenesis across the entire genome. Surprisingly, sequencing assay showed that our CRISPR/Cas9 system did not introduce significant off-target cleavages in the pig genome, though a high number of sgRNAs were employed in the present study. In additional, we also did not find any mosaic mutations in the piglets by whole-genome sequencing. It suggested that the choice of sgRNAs that represent unique sequences in the genome and the use of optimization of the injection method would suffice to avoid off-target mutations and reduce the mosaic rate and thus cut the cost and time required for the production of mutant animals.

We expect that the newly developed CRISPR/Cas9 technology will promote the generation of human diseases pig models and pigs with tailored economic traits, and yield results of high medical and agricultural value.

## Material and Methods

### Chemical and Reagents

Unless otherwise stated, all chemicals were purchased from Sigma (St. Louis, MO, USA).

### Animals and Ethics statement

Pigs were raised at the Beijing Farm Animal Research Center (attached to Institute of Zoology, Chinese Academy of Sciences) and had ad libitum access to a commercial pig diet (nutrient levels according to the NRC) and water throughout the experimental period. All experiments involving animals were conducted according to the Guidelines for the Care and Use of Laboratory Animals established by the Beijing Association for Laboratory Animal Science and approved under the Animal Ethics Committee of Institute of Zoology, Chinese Academy of Sciences.

### Generation of *DJ-1/parkin/PINK1* triple-gene modified pigs using CRISPR/Cas9 system

Production of Cas9 mRNA and sgRNA, recovery of *in-vivo*-derived zygotes, cytoplasmic microinjection of RNAs, embryo transfer and Sanger sequencing of mutated sites were performed as described in our previous report^24^. The primers used in producing the PCR amplification for sgRNA *in vitro* transcription and detecting the mutagenesis at the targeted site were listed in Supplemental Tables S3.

### Preparation of porcine ear fibroblasts and culture conditions

Pig ear fibroblast cells were prepared from ear skin biopsies from wild type or gene modified miniature pigs. The fibroblasts were cultured in DMEM, supplemented with 15% defined FBS, 1 × non-essential amino acids, 100 units/mL penicillin, and 100 μg/mL streptomycin in a humidified atmosphere containing 5% CO_2_ at 37 °C.

### RNA isolation and real-time RT-PCR

Total cellular RNA (tcRNA) was extracted from wild type or gene modified miniature pig fibroblasts using a RNAprep pure tissue kit (TIANGEN Biotech, Beijing, China). Real-time reverse transcriptase polymerase chain reaction (RT-PCR) was conducted using an ABI ViiA™ 7 system (Applied Biosystems, Foster City, CA, USA) and SYBR Green as the double-stranded DNA-specific fluorescent dye (Bio-Rad, Hercules, CA, USA) (see Supplementary Table S3 for RT-PCR primer sets). The pig GAPDH gene was used as an internal control to normalize the RT-PCR efficiency and to quantify the expression of the genes. After normalization with actin mRNA, we compared the relative expression of each mRNA in the wild type and two gene modified miniature pig-derived genes with those of the controls. We performed RT-PCR on each sample independently and in triplicate.

### Immunofluorescence

Pig ear fibroblast cells were seeded into 24-well plates (BD Falcon, Franklin Lakes, NJ). On the next day, the cells were rinsed with 1X PBS and fixed with 4% paraformaldehyde for 10 min at room temperature followed by permeabilization with PBS plus 0.1% Triton X-100. The cells were subjected to immunofluorescence staining with a DJ-1 antibody (1:500, Cell Signaling Technology, Inc. MA, USA) overnight at 4 °C. The cells were then washed with cold PBS three times for 3 min per wash and incubated with a FITC-labeled anti-rabbit secondary antibody (1:800) (Life Technologies, Carlsbad, CA, USA) at room temperature for 1 h. After incubating the cells with 0.1 μg/ml DAPI (DNA stain) for 1 min followed by rinsing with PBS, the cells were analyzed by confocal microscopy using a LSM 5 PASCAL (ZEISS, Oberkochen, Germany).

### Whole genome sequencing

Genomic DNA was extracted from the ear tissue of each pig, and whole genome sequencing was performed using an Illumina NextSeq 500 system according to the manufacturer’s instructions. For each individual (boar, sow and offspring), index sequence was added and a 2 × 150 paired-end sequencing library with 300 bp insert size was generated. The three libraries were then pooled together and sequenced in a single run with a mean coverage of 11X (boar, 10.2X; sow, 11.2X; offspring, 11.7X.). The original sequence reads were split based on index and the adapters were trimmed out. A trimmomatic program was further used to remove adapter contamination and trim sequencing reads with low quality bases with the command ‘illumina_adapters.fa:2:30:10 LEADING:3 TRAILING:3 SLIDINGWINDOW:4:15 MINLEN:50’. The remaining qualified reads were then mapped to pig build 10.2 reference sequence using Burrows-Wheeler Aligner (BWA) tools with default parameters. The SAM files (generated from BWA) that contained the read alignments were converted into BAM files, and the processed BAM files (sorting and removing duplicates) were then used to call variants by SAMtools program. The BEDTools software package was used for analyzing the coverage distributions. In the present whole genome sequencing analysis, about 70% of genomic sites (~37M) within the coding sequences (CDS) were covered by at least 10 sequencing reads (Fig. 3A and Supplemental Figure S1).

### De novo mutation calling and filtering

To investigate de novo insertion/deletions (indels) mutations (introduced by off-targeting) on a genome-wide scale using trio-based sequencing data, a trio calling algorithm implemented in VarScan software was applied with the following parameters as ‘–min-coverage 10 –min-reads2 2 –min-var-freq 0.3 –min-avg-qual 20 -adj-var-freq 0.05 -adj-p-value 0.1’. To further filter the de novo mutations, a series of stringent parameters were used to reduce the rate of false positive: (a), no variant-supporting reads in either parent. (b), genotype quality (GQ) score of the variant should be higher than 25. (c), variants in the repetitive regions of pig genome were excluded. (Information for repetitive elements in pig genome was downloaded from UCSC table browser.)

### Prediction of off-target sites

Putative off-target sites were investigated using the CasOT program, and the maximum number of mismatches allowed in the seed region and non-seed region of potential off-target sites were limited to 2. To examine whether the potential off-target sites were located in exonic regions, annotation GTF file of pig gene-set (downloaded from Ensembl database) was provided.

## Additional Information

**How to cite this article**: Wang, X. *et al*. One-step generation of triple gene-targeted pigs using CRISPR/Cas9 system. *Sci. Rep*. **6**, 20620; doi: 10.1038/srep20620 (2016).

## Supplementary Material

Supplementary Information

## Figures and Tables

**Table 1:**
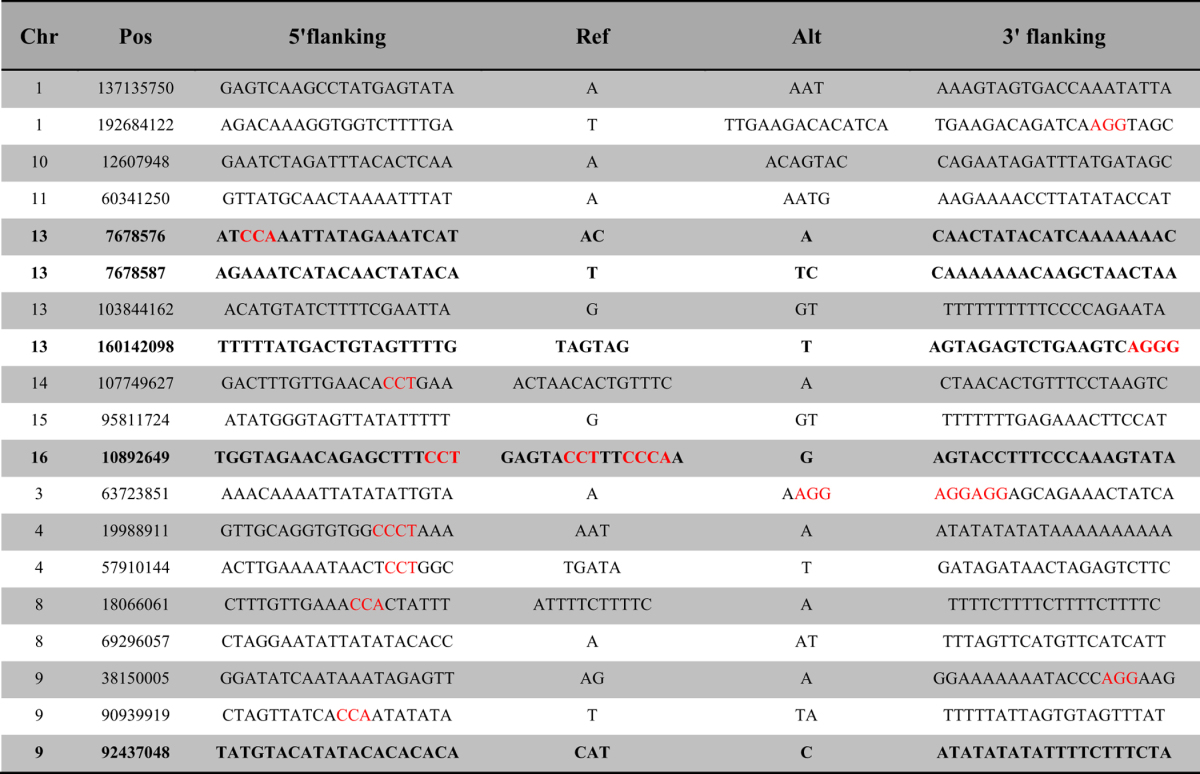
Unique de novo indels detected by whole-genome sequencing.

No indels lie in the coding sequences. Indels that lie in the introns are highlighted in Bold. Other indels lie in the intergenic region. Red highlights indicate potential PAMs (NGG) within 20 bases upstream of the indel.
