# Supplementary material for: One-step generation of triple gene-targeted pigs using CRISPR/Cas9 system
Source: Sci Rep. 2016 Feb 9;6:20620. doi: 10.1038/srep20620 (PMC4746670; doi:10.1038/srep20620)
Supplement: Supplementary Information [file srep20620-s1.pdf]

Supplemental Information

One-step generation of triple gene-targeted pigs using CRISPR/Cas9 system

Xianlong Wang[1,5], Chunwei Cao[1,5], Jiaojiao Huang[1,5], Jing Yao[1],Tang Hai[1], Qiantao Zheng[1,2], Xiao Wang[1,2], Hongyong Zhang[1,2], Guosong Qin[1], Jinbo Cheng[4], Yanfang Wang[3], Zengqiang Yuan[4], Qi Zhou[1,2], Hongmei Wang[1,2*], Jianguo Zhao[1,2*]

[1]State Key Laboratory of Stem Cell and Reproductive Biology, Institute of Zoology, Chinese Academy of Sciences, Beijing 100101, China

[2]University of Chinese Academy of Sciences, Beijing 100049, China

[3] Institute of Animal Sciences, Chinese Academy of Agricultural Sciences, Beijing 100193, China

[4]State Key Laboratory of Brain and Cognitive Sciences, Institute of Biophysics, Chinese Academy of Sciences, Beijing 100101, China

[5]These authors contributed equally to this work.

[*] Corresponding author: J Zhao, zhaojg@ioz.ac.cn; H Wang, wanghm@ioz.ac.cn;

Contents:

1. Supplementary Figure S1
2. Supplementary Figure S2
3. Supplementary Table S1
4. Supplementary Table S2
5. Supplementary Table S3

Figure S1. Cumulative distribution of coverage of whole genome sequencing for three individuals. The graph indicates the fraction of bases in the coding sequence achieving coverage equal or higher than the coverage indicated on the x axis.

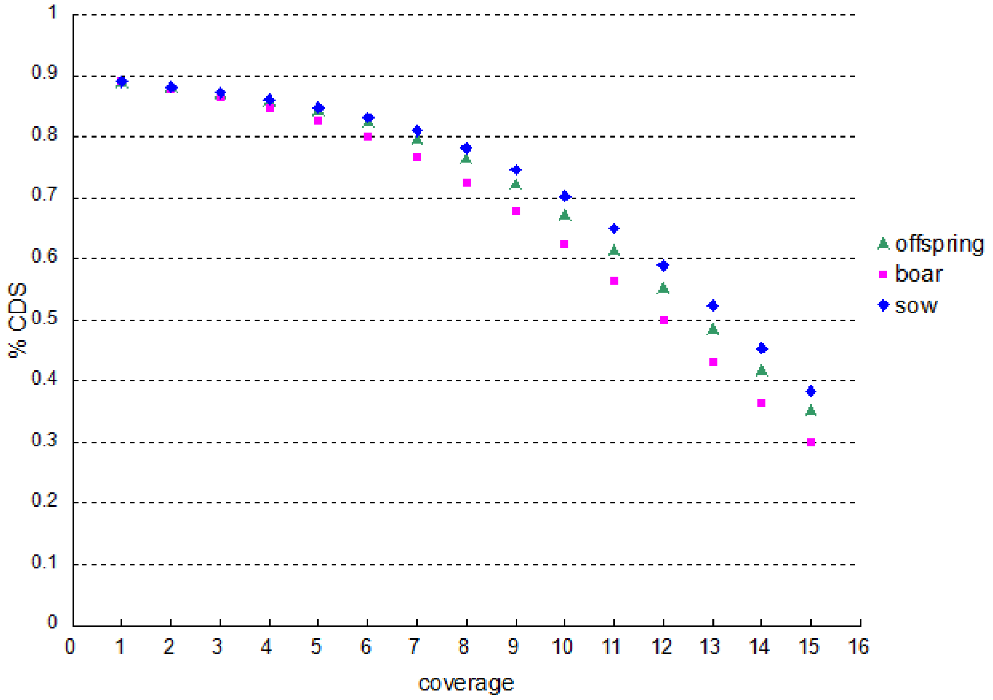

Figure S2. DNA Sanger sequencing of the 12 potential targeting sites in the coding regions (as indicated in Table S2) for the two gene-modified piglets. The red boxes indicate the presence of putative off-target sites in the electropherogram.

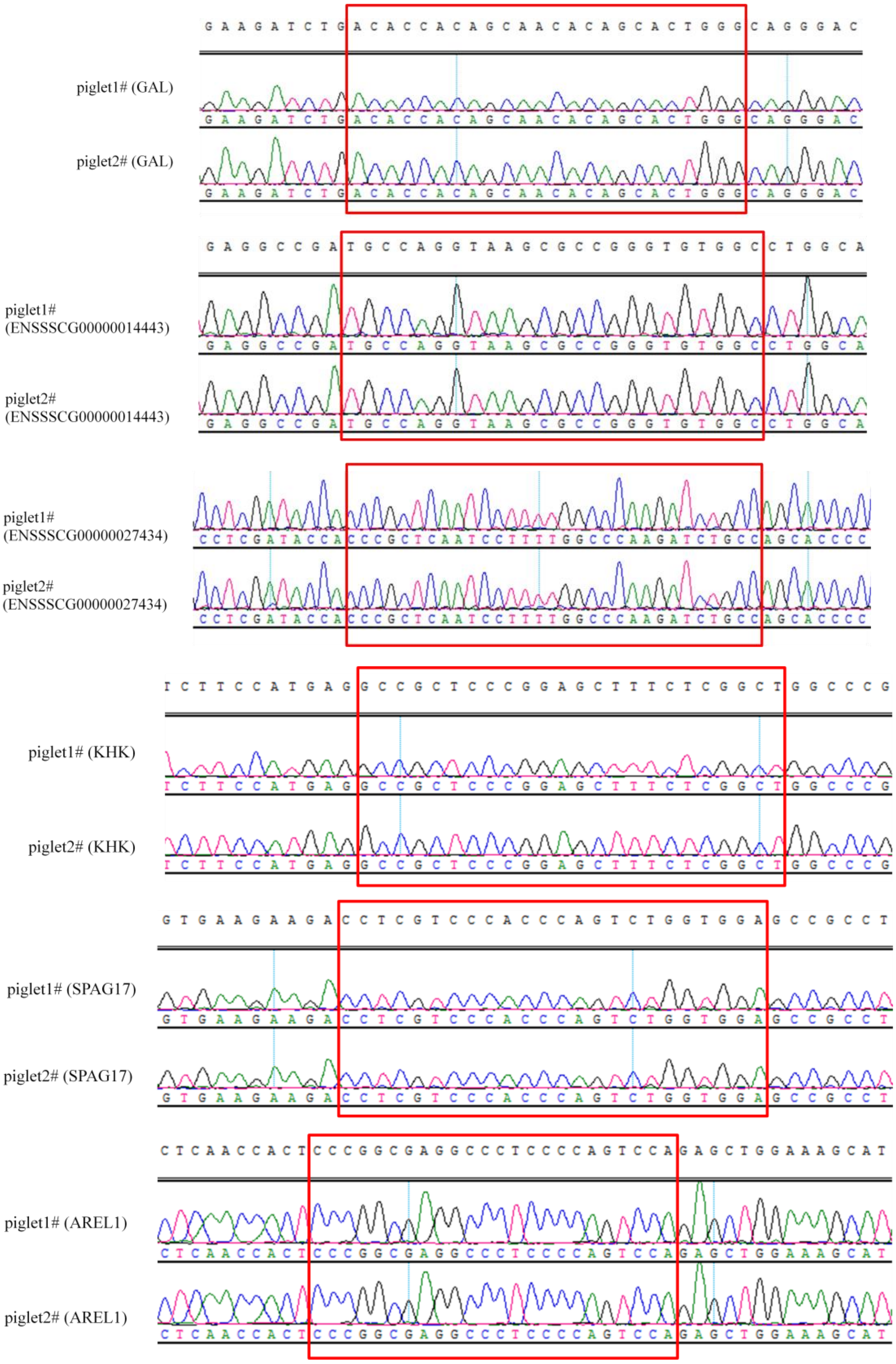

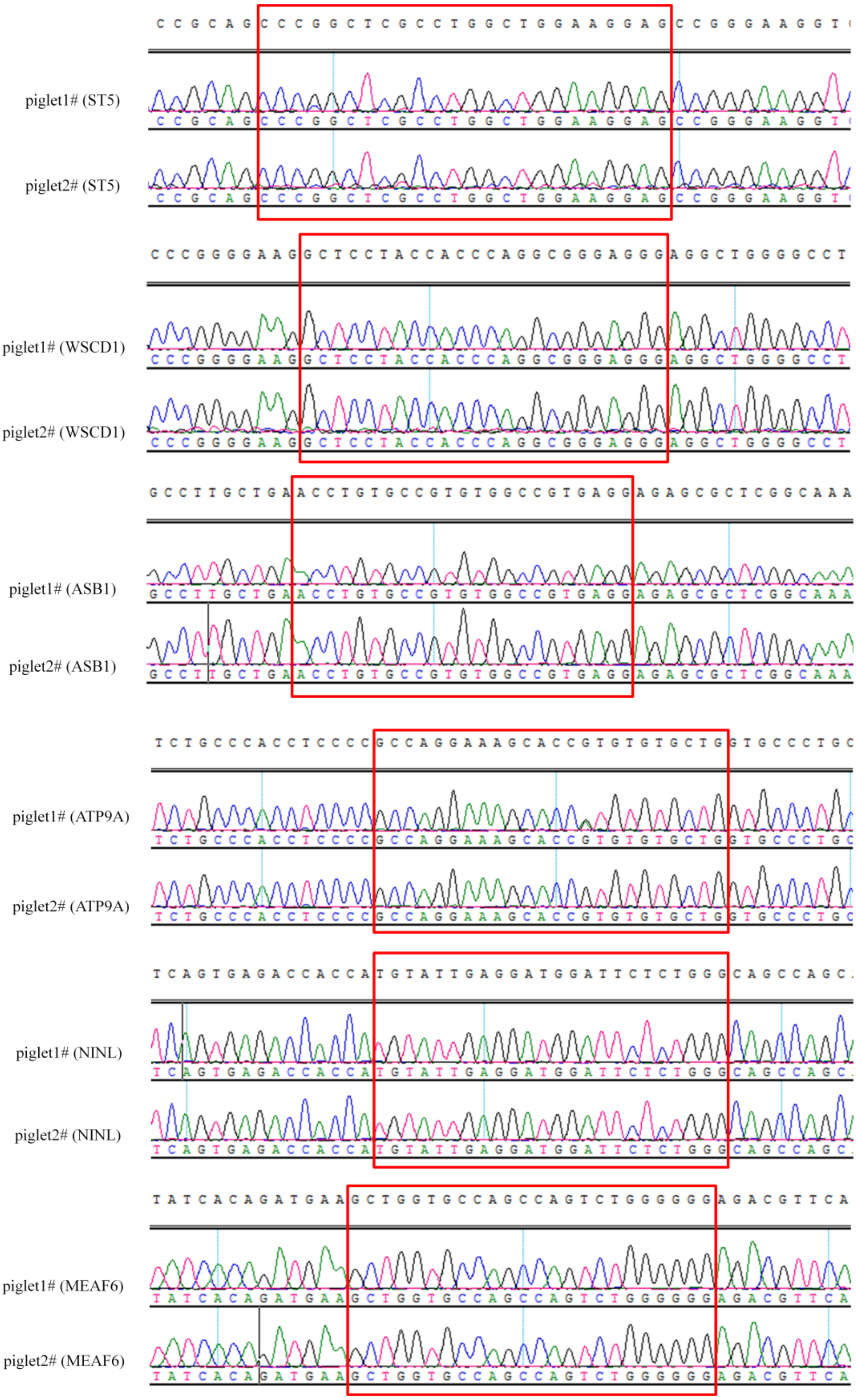
CCGCAGCCCGGCTCGCCTGGCTGGAAGGAGCCGGGAAGGT
piglet1# (ST5)
piglet2# (ST5)
CCCGGGGAAGGCTCCTACCACCCAGGCGGGAGGGAGGCTGGGGCCT
piglet1# (WSCD1)
piglet2# (WSCD1)
GCCTTGCTGAACCTGTGCCGTGTGGCCGTGAGGAGAGCGCTCGGCAAA
piglet1# (ASB1)
piglet2# (ASB1)
TCTGCCCACCTCCCCGCCAGGAAAGCACCGTGTGTGCTGGTGCCCTGC
piglet1# (ATP9A)
piglet2# (ATP9A)
TCAGTGAGACCACCATGTATTGAGGATGGATTCTCTGGGCAGCCAGC
piglet1# (NINL)
piglet2# (NINL)
TATCACAGATGAAGCTGGTGCCAGCCAGTCTGGGGGGAGACGTTCA
piglet1# (MEAF6)
piglet2# (MEAF6)

Table S1. Summary of embryo microinjection of Cas9 mRNA and sgRNAs and embryo transfer.

	Transferred zygotes	Newborns	Mutant	Triple mutant (% of Newborns)
Experiment 1	7	0	0	0
Experiment 2	17	2	2	2 (100%)
Experiment 3	10	0	0	0

Table S2. Sequencing depth of putative off-target sites in the coding regions.

Chr	Start	End	Genes	offspring	Sire	Dam
**2**	2931403	2931426	GAL	15.7X	11.4X	15.1X
**2**	158144934	158144957	ENSSSCG00000014443	20.8X	21.3X	9.0X
**2**	69135434	69135457	ENSSSCG00000027434	5.8X	8.7X	10.0X
**3**	119029646	119029669	KHK	27.7X	12.7X	20.4X
**4**	112589288	112589311	SPAG17	15.7X	20.9X	15.2X
**7**	103746297	103746320	AREL1	12.0X	14.2X	13.9X
**9**	682525	682548	ST5	8.7X	8.6X	6.0X
**12**	53261323	53261346	WSCD1	8.2X	11.7X	13.8X
**15**	152397991	152398014	ASB1	8.6X	12.8X	22.2X
**17**	59380024	59380047	ATP9A	8.6X	18.6X	14.7X
**17**	35258110	35258133	NINL	12.4X	11.0X	10.5X
**GL895879.1**	1609	1632	MEAF6	13.6X	12.8X	14.3X

Table S3. Oligonucleotides used in this study. Underlines indicate the sgRNA targeting sites.

**Oligonucleotides for genotyping**	
Primer name	Sequence
DJ-1 F	CAGACAGAGTGCTGGGACAG
DJ-1 R	TGAATCACACAACCACGCCT
Parkin F	ATAAGTCACAGGGCCGTTGG
Parkin R	AAAGAAACGGGGGCAATGAC
PINK1 F	AAACTAGGCTTGGCTTGCCC
PINK1 R	GAGGGCACAGGTGAGGATTC
**Oligonucleotides for *in vitro* transcription**	
Primer name	Sequence
sgDJ1-1 F	TAATACGACTCACTATAGGACATCACGGCTACACTGCACGTTTTAGAGCTAGAAATAGC
sgDJ1-2 F	TAATACGACTCACTATAGGCATTTGTCCCGACGCCAGCCGTTTTAGAGCTAGAAATAGC
sgparkin-1 F	TAATACGACTCACTATAGGCACCGCACGCGGAGCTTTCCGTTTTAGAGCTAGAAATAGC
sgparkin-2 F	TAATACGACTCACTATAGGAGCCAGGAAAGCTCCGCGTGGTTTTAGAGCTAGAAATAGC
sgPINK1-2 F	TAATACGACTCACTATAGGTGTACTGAGGATGGCTTCGCGTTTTAGAGCTAGAAATAGC
sgPINK1-2 F	TAATACGACTCACTATAGGGCTCGTCCCAGCCAGTCGGGGTTTTAGAGCTAGAAATAGC
sgRNA R	AGCACCGACTCGGTGCCACT
**Oligonucleotides for real-time PCR**	
Primer name	Sequence
DJ-1 rt F	AGAGGAAGGGCCTCATAGCA
DJ-1 rt R	AGTGGGTGCGTCGTAACTTT
Parkin rt F	CCAAACCGGATGAGTGGTGA
Parkin rt R	CTTGTCAGAGGTCGGGTGTG
PINK1 rt F	CTCTGGTCGACTACCCCGAT
PINK1 rt R	ATGACGAGGAAGAGTGTCCG
PRDX2 F	GGCCTTCCAGTACACAGACG
PRDX2 R	TCCACGTTGGGCTTGATTGT
GCLM F	AGTGGGCACAGGTAAAACCA
GCLM R	AGTTAAATCGGGCGGCATCA
NQO1 F	GATCATACTGGCCCACTCCG
NQO1 R	CATGGCATACAGGTCCGACA
TRX1 F	ACGCTTTTCAGGAAGCCTTG
TRX1 R	TCATTTTGCAAGGCCCACAC
KEAP1 F	GAGGTGGTGTCCATTGAGGG
KEAP1 R	GAGCACACACTTCTCACCCA
GAPDH F	ACCAGGTTGTGTCCTGTGAC
GAPDH R	AGCTTGACGAAGTGGTCGTT
**Oligonucleotides for putative off-target sites in the coding regions**	
GAL	F: CTCTGTGCGCCTCATTATCA
	R: GGCAGCATCCTCAGAAGAC
ENSSSCG00000014443	F: GGGCTGAGGTTCTGAGGTC
	R: CCACTCAGGGACGCCAAC

ENSSSCG00000027434	F: AGGTTGTCCCCCAAACCATC
	R: AATTGACAACCACGTGACGGA
KHK	F: AATGCACTCAAGGCCACAG
	R: CAGCCTGCTGAGCGAAAG
SPAG17	F: GAAACTCCCTTGTATTTATGAAAAGC
	R: ACGCCCTCTGACCTTTTTCT
AREL1	F: TGTCACTGAATCGGGTGAAG
	R: GGGTGGAGTGGAGGACACTA
ST5	F: CGTCTCCATGACGACCGGC
	R: GCCAGGGAAGGAGGAGAGAA
WSCD1	F: CGTGAGGAAGAAGAGCAGGA
	R: AGGGACAGTAACCCCAGGAG
ASB1	F: GAGAGTGAGGCGAGGAGGT
	R: CTCCGGCTCTGGAATCCTAC
ATP9A	F: CTTACGGTGTCCAGGCTGTC
	R: CCGGAGTCACAGAGAAGGTC
NINL	F: TAATGCCCAGTAGGGTCCTG
	R: GTGCGAAGAGCAAGAGCTG
MEAF6	F: AGCCTCCCTCTGAAATGTGA
	R: AGACCACATCGGATCATTCA
